# Clinicopathologic Characteristics and Outcomes of Histiocytic and Dendritic Cell Neoplasms: The Moffitt Cancer Center Experience Over the Last Twenty Five Years

**DOI:** 10.3390/cancers6042275

**Published:** 2014-11-14

**Authors:** Samir Dalia, Michael Jaglal, Paul Chervenick, Hernani Cualing, Lubomir Sokol

**Affiliations:** 1 Mercy Clinic Oncology and Hematology-Joplin, 3001 MC Clelland Park Blvd, Joplin, MO 64804, USA; 2 Department of Malignant Hematology, H. Lee Moffitt Cancer Center, 12902 Magnolia Drive, Tampa, FL 33602, USA; E-Mails: Michael.jaglal@moffitt.org (M.J.); paul.chervenick@moffitt.org (P.C.); 3 IHCFLOW Histopathology Laboratory, University of South Florida, 18804 Chaville Rd., Lutz, FL 33558, USA; E-Mail: IHCFLOW@verizon.net

**Keywords:** dendritic cell tumor, Langerhans cell histiocytosis, histiocytic sarcoma, follicular dendritic cell sarcoma, interdigitating dendritic cell sarcoma, indeterminate dendritic cell sarcoma, fibroblastic reticular cell tumor

## Abstract

Neoplasms of histiocytic and dendritic cells are rare disorders of the lymph node and soft tissues. Because of this rarity, the corresponding biology, prognosis and terminologies are still being better defined and hence historically, these disorders pose clinical and diagnostic challenges. These disorders include Langerhans cell histiocytosis (LCH), histiocytic sarcoma (HS), follicular dendritic cell sarcoma (FDCS), interdigtating cell sarcoma (IDCS), indeterminate cell sarcoma (INDCS), and fibroblastic reticular cell tumors (FRCT). In order to gain a better understanding of the biology, diagnosis, and treatment in these rare disorders we reviewed our cases of these neoplasms over the last twenty five years and the pertinent literature in each of these rare neoplasms. Cases of histiocytic and dendritic cell neoplasms diagnosed between 1989–2014 were identified using our institutional database. Thirty two cases were included in this analysis and were comprised of the following: Langerhans cell histiocytosis (20/32), histiocytic sarcoma (6/32), follicular dendritic cell sarcoma (2/32), interdigitating dendritic cell sarcoma (2/32), indeterminate dendritic cell sarcoma (1/32), and fibroblastic reticular cell tumor (1/32). Median overall survival was not reached in cases with LCH and showed 52 months in cases with HS, 12 months in cases with FDCS, 58 months in cases with IDCS, 13 months in the case of INDCS, and 51 months in the case of FRCT. The majority of patients had surgical resection as initial treatment (*n* = 18). Five patients had recurrent disease. We conclude that histiocytic and dendritic cell neoplasms are very rare and perplexing disorders that should be diagnosed with a combination of judicious morphology review and a battery of immunohistochemistry to rule out mimics such as carcinoma, lymphoma, neuroendocrine tumors and to better sub-classify these difficult to diagnose lesions. The mainstay of treatment for localized disease remains surgical resection and the role of adjuvant therapy is unclear. In patients with multiple areas of involvement, treatment at tertiary care centers with multimodality treatment is likely needed. Accurate subset diagnosis will contribute to better data as well as treatment outcomes analysis of these rare disorders of adult patients in the future.

## 1. Introduction and Methods

### 1.1. Introduction

Tumors of histiocytes and dendritic cells are rare hematopoietic neoplasms which together make up less than one percent of all neoplasms occurring in the lymph nodes or extramedullary locations. Recently, these tumors have been further classified by origin into either mesenchymal cells or bone marrow precursor-derived neoplasms [1,2]. Langerhans cell histiocytosis (LCH), histiocytic sarcoma (HS), and interdigitating dendritic cell sarcoma (IDCS) are conventionally considered to be derived from bone marrow precursors. Follicular dendritic cell sarcoma (FDCS), fibroblastic reticular cell tumors (FRCT), and indeterminate dendritic cell sarcoma (INDCS) originate from putative mesenchymal stromal tissue [1,2].

Historically, tumors of histiocytic and dendritic cell origin have been difficult to diagnose due to their histological similarities with non-Hodgkin lymphoma, melanoma, sarcoma, and multiple undifferentiated carcinomas. With recent advancements in immunohistochemistry, new insights into their tumor biology, clinical features, and treatment have been reported for these rare neoplasms. Due to the rarity and wide age presentation of these disorders much of the published literature either reports data in both pediatric and adult patients or otherwise, is comprised of isolated case reports with literature reviews. Little is known about optimal treatment in adult patients with these rare disorders. We believe that when possible, patients should be seen at centers that have specialists who have seen and treated patients with these rare diseases.

Differential diagnosis of patients with histiocytic and dendritic cell tumors includes B and T cell lymphomas, melanoma, metastatic sarcoma, and metastatic carcinoma. Due to the broad differential options, many of these tumors are diagnosed as some other entity. A collaboration between surgical pathology, who often see these cases, and hematopathology including morphologic review and immunohistochemistry are of utmost importance to accurately diagnose these “orphan” diseases. In order to gain a further better understanding of the biology, diagnosis, and treatment in these rare disorders, we reviewed cases of these neoplasms in our center seen over the last twenty five years and summarized the pertinent literature in each of these rare neoplasms.

### 1.2. Methods

After obtaining institutional board review approval, we identified all cases of dendritic cell and histiocytic tumors seen at the H Lee Moffitt Cancer Center (Tampa, FL, USA) between 1 January 1989 and 31 March 2014 using our institutional database. Prior to 2008 and in cases in which pathology was done outside our institution and further tissue was not available, diagnoses were made by review of histopathology by at least two academic pathologists. Immunohistochemical (IHC) staining based on criteria used in the WHO 2008 classification of hematopoietic neoplasms was used when tissue was available to aid in diagnosis [2]. Clinical data including age, sex, locations of tumors and survival data was collected. Treatment data including initial chemotherapy, radiation therapy, surgery, or skin directed therapy and if the patient had relapsed were also recorded.

Statistical analysis comprised of reporting of descriptive statistics. Overall survival was defined as time from initial diagnosis to time of death. Overall survival was calculated using the Kaplan-Meier method. One patient was lost to follow-up at time of the diagnosis and thus does not have any survival data and was censored. Due to the small sample size of each individual disease, univariate modeling for prognostic factors could not be obtained. Statistical analysis was performed using SPSS version 21 (IBM Corp., Armonk, NY, USA, 2012).

## 2. Results

Our search resulted in a total of thirty eight cases. Six cases were excluded because they were not correctly classified and were not dendritic cell or histiocytic neoplasms (*n* = 5) and one did not have any information in the medical or pathological record for evaluation (*n* = 1). Thirty two cases were included in this analysis and comprise the following diagnoses: Langerhans cell histiocytosis (LCH) (20/32), histiocytic sarcoma (6/32), follicular dendritic cell sarcoma (2/32), interdigitating dendritic cell sarcoma (2/32), indeterminate dendritic cell sarcoma (1/32), and fibroblastic reticular cell tumor (1/32). Clinical and pathological characteristics of the thirty two cases are reported in Table 1.

### 2.1. Langerhans Cell Histiocytosis

Twenty cases were diagnosed with LCH. The median age was 36 (Range 3–71) years and the majority were female (*n* = 13). The majority had prolonged survival with the median survival not being reached at the time of analysis. Seventy-three percent of cases were alive at 75 months and only three had a recurrence of their initial tumor. Staging CT scans were done in 80% of cases and staging bone marrow biopsy was done in 55%. Initial presentation included those from lungs, bone, skin, lymph nodes, bone marrow and rarely from brain, pituitary and parotid gland.

**Table 1:** Characteristics of 32 cases of dendritic cell and histiocytic neoplasm.

Case	Year of Diagnosis	Age at Diagnosis (Year)/Sex	Disease Subtype	Initial Site of Involvement	Initial Staging CT Scans/Bone Marrow (Yes/No)	Recurrence (Yes/No)	Immunohistochemistry	Vital Status	Overall Survival (months)
1	2010	18/F	LCH	Lungs/Mandible	No/No	No	NR	Alive	41
2	2011	26/M	LCH	Lungs/Skin (nose)	Yes/No	No	Cd1a, CD68, S100	Alive	6
3	2011	16/F	LCH	Right Femur	No/No	No	CD1a, S100, CD68	Alive	20
4	2008	26/F	LCH	Lung/Bone/C2 spine	Yes/No	NR	CD1a, vimentin, S100, CD68, CD31 (focal), LCA (focal) (CD20-, CD30-, Cd99-, CD117-, melan-A-)	Alive	30
5	2012	53/F	LCH	Right temporal bone	Yes/No	No	CD1a, S100	Alive	14
6	2010	51/F	LCH	Skin (nose)	Yes/Yes	NR	CD1a, (HMB-45-, CD117-, CD45-, CD3-, CD20-)	Alive	1
7	2007	22/M	LCH	Ischial tuberosity	Yes/Yes	Yes	CD1a, CD68, S100 (CD3-, CD20-, CD5-, Cd15-, CD30-, PAX5-, MUM1-)	Alive	77
8	2011	59/F	LCH	Skin (buccal)	Yes/No	No	CD1a, S100, CD43 (CD20-, CD3-,Cd30-, MPO-, HMB-45-, CK 5/6-, P63-)	Alive	20
9	2008	60/F	LCH	Cecum/Lymph Nodes	Yes/Yes	No	CD1a, CD68, S100 (melan-A−, CD117−, HMB45−)	Alive	64
10	2005	17/M	LCH	Lung/Bone/Pituitary/Hypothalamus	Yes/No	No	CD1a, S100	Dead	75
11	2007	42/F	LCH	Lung/T3	Yes/Yes	Yes	CD1a, S100, CD68	Alive	61
12	2009	38/F	LCH	Parotid Gland	Yes/Yes	No	CD1a, CD68, S100	Dead	12
13	2008	61/M	LCH	Lymph Nodes/Inguinal lymph node	Yes/Yes	No	CD1a, CD4, S100, Langerin, CD68 weak positive, CD30 weak positive, Ki-67 30%−40%, (Alk-1−, panmelanoma−, oscar−, CD20−, CD21−, CD23−)	Alive	3
14	2005	25/F	LCH	Skin (scalp)	Yes/Yes	No	CD1a, CD68, S100	Alive	102
15	1996	25/F	LCH	Right frontal lobe	Yes/Yes	No	NR	Alive	191
16	2011	33/F	LCH	Skin (shoulder, face)	Yes/No	No	CD1a, S100, (-CD68, -melanA)	Alive	26
17	2007	43/M	LCH	Seventh rib	No/No	No	CD1a, CD68, S100 (Pankeratin−)	Alive	76
18	2009	71/F	LCH	Lung/bone marrow	Yes/Yes	No	CD1a, S100, (CD68−, lysozyme−, MPO−)	Dead	9
19	2012	55/M	LCH	Skin (arm, back)	Yes/Yes	No	CD1a, CD68, S100, (CD 34−)	Alive	5
20	1985	3/M	LCH	Bone marrow/lymph node	NR/Yes	Yes	NR	Alive	347
21	2005	69/F	HS	Intestine	Yes/No	Yes	CD4, CD45RO, CD45, CD68, Vimentin, CD163 (S100−)	Dead	52
22	2013	52/M	HS	Brain	Yes/No	No	NR	Dead	5
23	1995	56/F	HS	Bone marrow	NR/Yes	No	NR	Alive	221
24	2003	51/F	HS	Neck lymph node	Yes/No	No	CD68, Vimentin, Lysozyme, S100 (LCA−, CD30−, CK20−, CAM5.2−, TTF-1−, HMB-45−, CD34−, SMA−)	Alive	101
25	1992	51/M	HS	Skin (thumb)	NR/NR	No	S100(−), LCA(−), Vimentin		
26	1994	68/M	HS	Bone marrow	NR/Yes	NR	NR	Dead	0
27	2005	42/M	FRCT	Inguinal lymph node/nodes	Yes/Yes	No	CD14, S100, CD45, SMA (CD21−, CD35−, CD20−, CD10−, CD3−, AE/CAM5.2−)EMA	Alive	51
28	2008	77/M	FDCS	Abdominal mass	Yes/NR	Yes	CD 21, CD23, CD68, D-240 (CD117−, desmin−, S-100−)	Dead	22
29	2014	46/F	FDCS	Liver and lymph nodes	Yes/No	No	CD21, CD23, CD68 weak, S100+, CD138−, AE1/AE/CAM5.2−	Alive	2
30	2008	90/F	IDCS	Soft tissue (anterior chest)	NR/NR	NR	CD68, S100, Vimentin (CD15−, CD30−, CD20−, CD3−, MART-1−, CD34−)	NR	NR
31	2008	50/M	IDCS	Cervical lymph node	Yes/Yes	No	S100, Vimentin, CD68 (CD35−, CD21−, CD3−, CD20−, CD34−, SMA−, AE1/AE3−, CAM5d3−, Melan-A−, Ki low).	Alive	58
32	2008	45/F	INDCS	Skin (supraclavicular)	Yes/Yes	No	CD1a, CD68, S100 (CD30−)	Alive	13

M: male, F: female, LCH: Langerhans cell histiocytosis, HS: histiocytic sarcoma, FDCS: Follicular dendritic cell sarcoma, IDCS: Interdigitating dendritic cell sarcoma, INDCS: Indeterminate dendritic cell sarcoma, FRCT: Fibroblastic reticular cell tumor, NR: Not reported, SMA: smooth muscle actin.

The majority of cases were treated with surgical resection at diagnosis (*n* = 12). Three patients received skin directed therapy which included topical steroids (Case 14), and psoralen + ultraviolet light-A (PUVA) (Cases 16, 19). Three cases initially received involved field external radiation therapy (Cases 4, 7, 13). Systemic therapy was given initially in four cases (Case 2, 10, 11, 13) and included prednisone (Case 2), vinblastine and prednisone followed by 2-CDA (Case 10), on a clinical trial protocol (Case 11), and cyclophosphamide, doxorubicin, vincristine, prednisone (CHOP) (Case 13). At relapse, three cases received chemotherapy which included vinblastine and prednisone followed by 6-mercaptopurine and prednisone (Case 7), methotrexate and cytarabine (Case 11), and methotrexate and vinblastine (Case 20). Case 10 developed therapy-related myelodysplastic syndrome and completed allogeneic stem cell transplantation but eventually succumbed to transplant related complications. Case 18 developed acute myelogenous leukemia (not phenotypically related to Langerhans cells) concurrently and died.

Immunohistochemistry (IHC) was positive for CD1a, S100, and CD68 in tumors of 10 of these patients. B and T cell markers were negative in all cases with IHC data. Three patients did not have IHC data reported in their pathological records and these were irretrievable blocks. Langerin expression was not done in the majority of cases and because most initial diagnoses were done at other institutions this test could not be rerun. 

### 2.2. Histiocytic Sarcoma

Six cases were diagnosed with histiocytic sarcoma. The median age was 54 (51–69) years and three were female. The median overall survival was 52 months (Range 0–153 months) and two cases had a recurrence. Staging with CT scans was completed in 50% of cases and bone marrow biopsy was completed in 33% of cases. Initial presentation included involvement of bone marrow, brain, intestine, lymph node, and skin. 

Initial treatment consisted of surgery followed by CHOP chemotherapy (Case 21), surgery followed by concurrent cisplatin and radiation therapy (Case 24), and surgery followed by high dose methotrexate and cytarabine chemotherapy and radiation therapy (Case 22). One case surgical resection only (Case 25), one case received CHOP and etoposide chemotherapy (Case 23), and one received vincristine and cyclophosphamide therapy (Case 26). The morphologic hallmarks in reports were descriptive of histiocytic sarcoma after exclusion of other mimics such as carcinoma or lymphoma. IHC for histiocytic markers were not available for three cases. In the other cases S100 was negative in two cases and positive in one. Lysozyme, vimentin, and CD68 were positive in all cases with histological information. Due to limited tissue, we were unable to perform further immunohistochemical testing on some of our cases of HS and relied on morphological characteristics in order to make a diagnosis.

### 2.3. Follicular Dendritic Cell Sarcoma

Two cases had the diagnosis of FDCS. The median age was 62 (46−77) years, a female and a male. The median survival was 12 months (Range 2–22 months). One case was recently diagnosed and has only been followed for two months. Both cases had staging CT scans and only one had a staging bone marrow biopsy. Case 28 presented with an abdominal mass and case 29 presented in the liver and lymph node. Interestingly, case 29 was diagnosed in a patient also with prior Castleman’s disease.

Case 28 was treated with surgical resection followed by radiation therapy and CHOP chemotherapy for six cycles. Case 28 relapsed but did not receive any therapy at time of relapse. Case 29 was recently diagnosed and is currently undergoing treatment with radiation to the liver lesions.

IHC testing revealed CD21, CD23, CD68 positive tumors in both cases. S-100 was variable with one case being positive and one negative. 

### 2.4. Interdigitating Dendritic Cell Sarcoma

Two cases were diagnosed with IDCS. One case was male and median age was 70 (50–90) years. Clinical data was not available for case 30 except that the tumor was found in the soft tissue of the anterior chest. Case 31 had a survival of 58 months, received staging CT scans and bone marrow biopsy at diagnosis and was alive at last follow up. The initial diagnosis was made in a single cervical lymph node. Initial therapy in this case was with surgical resection. There were the typical morphology for IDCS and IHC testing showed S100, CD68, and vimentin positive tumors in both cases.

### 2.5. Indeterminate Dendritic Cell Sarcoma

One case had INDCS in our series (Case 32). This case was female and 45 years of age at diagnosis. Initial tumor was found in the skin of the supraclavicular region. She was staged with a bone marrow biopsy and CT scans of the neck, chest, abdomen, and pelvis and underwent treatment with surgical resection. She was lost to follow-up 13 months after diagnosis. By IHC staining her tumor was CD1a, CD68, and S-100 positive and negative for CD30.

### 2.6. Fibroblastic Reticular Cell Tumor

One case was diagnosed with FRCT (Case 27). The case was a male and was 42 years at diagnosis. The initial tumor was found in multiple lymph nodes and the patient was initially staged with a bone marrow biopsy and CT scans of the neck, chest, abdomen, and pelvis. He never underwent any treatment for his diagnosis and was monitored until 2009 (51 months post diagnosis) and was lost to follow-up. IHC revealed a tumor that was CD14, S100, and CD45, smooth muscle actin positive and negative for CD21, CD35, CD20, CD10, and CD3.

## 3. Discussion

In our series of thirty two cases of histiocytic and dendritic cell neoplasms we report that the majority of patients were diagnosed with localized LCH. HS, FRCT, FDCS, IDCS, and INDCS were rarely seen in our institution. The majority of these patients had localized disease and seventy seven percent of patients were alive at last follow up. Clinical presentation was variable in most patients and initial treatment was mainly surgical with radiation and chemotherapy used in select cases. Only one of our thirty two cases had an additional lymphoproliferative disorder that preceded the diagnosis (Case 29). All cases seen in our institution were reviewed at time of diagnosis by at least two hematopathologists using morphologic and immunohistochemical analysis. Those cases from outside institution for review that did have limited or inadequate immunostaining or staining positive for other mimics were not included in the series. In order to better understand these rare disorders we discuss the pertinent literature of each of these neoplasms. 

### 3.1. Langerhans Cell Histiocytosis

LCH represents a neoplastic transformation characterized as a clonal accumulation of Langerhans cells, a resident antigen present cell located in epidermis and dermis of the skin and other organs [3]. The majority of cases occur in childhood and unlike our adult cases, there is normally a male predilection [4]. LCH variant as a focal “eosinophilic granuloma” presentation has been described as a self-limiting disorder in the lungs of smokers, in which lesions may regress with the cessation of smoking [5].

Patients with suspected LCH are often asymptomatic and require a full staging work up. A comprehensive physical examination including that of the skin and visible mucous membranes is warranted. Complete blood count, blood chemistry, coagulation studies, thyroid stimulating hormone and free T4 should be obtained. Skeletal survey is warranted to assess for bone lesions and site specific CT scans are recommend on a case by case basis. The role of PET CT is controversial with a small study showing that PET-CT may identify lesions missed by other modalities and also can aid in documenting response to therapy [6]. In adults, bone marrow involvement is less likely and is only warranted in cases with specific symptoms including patients with B symptoms or cytopenias [5]. Patients should be stratified by single system LCH or multisystem LCH. In our twenty cases that presented multisystemically, 80% of patients had CT scans of involved sites and 55% had a bone marrow biopsy indicating that most patients had adequate staging work up. We did not see many skin limited LCH patients due to these being managed by dermatology. As a result, most of our patients had multiple involved sites since those patients warrant treatment by a hematologist/oncologist. 

**Figure 1:**
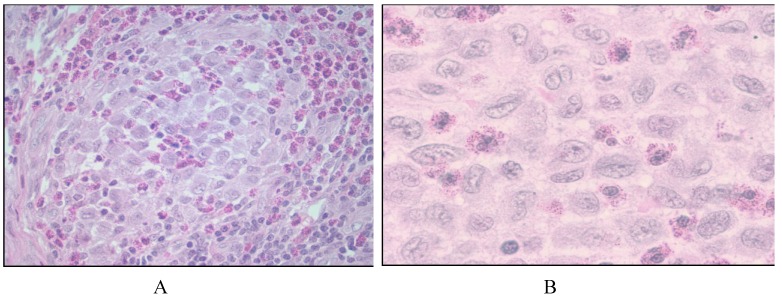
Langerhans cell histiocytosis. (**A**) Histologically, there is admixture of histiocytes, Langerhans cells, few multinucleated cells, small lymphocytes and eosinophils (left panel, 200×) and low mitotic figures; (**B**) Cytologically, Langerhans cells nuclei have histiocytic features with pale fine chromatin pattern and accentuated dented, folded or pronounced nuclear grooves, pale to pinkish generous circumferential cytoplasm with some degree of cytologic atypia without anaplasia (right panel, 1000×).

Histology of LCH reveals oval cells with grooved, folded, indented, or lobulated nuclei with areas of fine chromatin and pale to pinkish circumferential cytoplasm with cytologic atypia (Figure 1) [2]. Eosinophils are usually scattered throughout the infiltrate and in skin lesions, these are in the dermis and the epidermis is free of infiltrating cells [7].

Immunohistochemical staining reveals cells that are langerin and CD1a positive, both specific to LCH [3,8,9]. S100, CD45, CD101 and CD68 can be positive [2,9]. On ultra-structural examination Birbek granules are seen. 

Treatment of LCH can vary and depends on the clinical presentation. Patients with multisystem LCH, single system LCH with multifocal lesions, or single system LCH with certain presentations (central nervous system, liver, spleen) should be treated with systemic therapy. In cases where single system involvement is present in non-critical organs patients can be treated with excision, skin directed therapy (for skin only lesions) or radiation therapy. In patients with self-limited skin only disease treatment can include topical nitrogen mustard [10,11,12], psoralen plus ultraviolet A (PUVA) phototherapy [10,11], imiquimod [13], excimer laser [14] and radiation therapy [15] though most patients are initially treated with corticosteroids [16,17]. For bone only disease radiation therapy can be used if there is pain or impending fracture. In other cases observation is also appropriate [5].

In patients who require systemic therapy, agents such as methotrexate, cyclophosphamide, cyclosporine, 6-mercaptopurine, and vinblastine have been reported to have efficacy in LCH [11,17,18,19]. Generally, treatment should be based on the LC Histiocyte Society evaluation and treatment guidelines [20]. Currently, for multisystemic involvement, prednisone and vinblastine for 12 months is recommended [20,21,22]. Progressive disease or recurrence can be treated with 2-chlorodeoxyadenosine and cytarabine [23]. A recent case series has suggested that an inhibitor of mutated BRAF (vemurafenib) could be a novel promising targeted therapy for patients with LCH carrying BRAF^V600E^ mutations [24,25]. In our twenty cases, the majority of cases were treated with localized treatment with either surgical resection, skin directed therapy, or radiation therapy. Four cases had initial chemotherapy and two received chemotherapy for relapsed disease. One patient developed therapy related MDS and one patient had a concurrent diagnosis of LCH and AML. Only one patient was tested for BRAF^V600E^ mutation due to recurrent disease and was found to be negative. When possible patients should be treated at tertiary care centers where clinicians with better experience of treating LCH can be found. Collaborative efforts are needed to better understand prognosis in patients with LCH and to determine who would benefit from systemic therapy in this rare disease.

### 3.2. Histiocytic Sarcoma

HS is a rare non-Langerhans histiocyte disorder of mature tissue histiocytes. Associations between HS and with follicular lymphoma, myelodysplastic syndrome and acute lymphoblastic leukemia have been discovered [1,4,26,27]. This likely occurs through trans-differentiation from other hematological disorders. A study of patients with HS and follicular lymphoma reported the presence of t (14;18) and IGH gene rearrangements which was suggestive of a common clonal origin of follicular lymphoma and HS [28]. No of our cases of HS had another associated hematological disorder.

Similar to other reports, our median age was 54 years (reported range 46–55 years) and sex predilection has not been consistent but two small series reported a male predominance [4,26,29]. Rarely HS presents systemically or with multifocal disease [26,29,30]. The most common presentations are unifocal lesions in the intestines, skin, or soft tissue [4,26]. In our series three patients presented with intestine, skin, and single site nodal disease while the other three presented with bone marrow involvement, multi nodal disease, or brain involvement. Detailed physical examination including skin evaluation, staging CT scans to look for multifocal disease, and bone marrow biopsy are recommended to fully stage patients with HS. MRI of the brain should be done in cases in which clinical history or physical examination brings up a concern for brain involvement.

**Figure 2:**
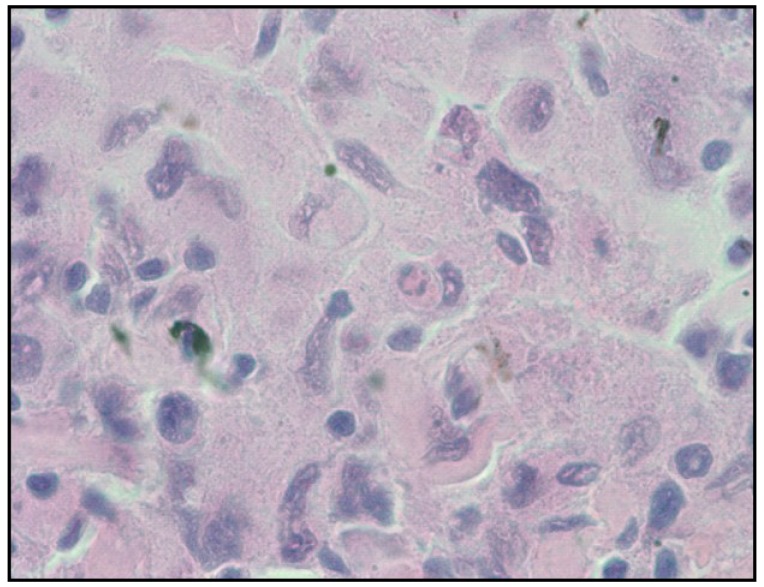
Histiocytic sarcoma. Histologic pattern showed a diffuse infiltrate with focal sinusoidal pattern by large atypical neoplastic cells with pleomorphic nuclei and moderate to abundant cytoplasm.

Non-cohesive proliferation of large cells that may have a focal sinusoidal pattern is seen on histological examination [4]. Nuclei are pleomorphic and can be eccentric and have one or more nucleoli. The cells may on occasion have a xanthomatous appearance (Figure 2) [2,4,26]. Immunochemistry is positive for histiocytic markers including CD163, CD68, and lysozyme while CD1a, CD21, CD35 are negative.

Much of what is known about treatment in HS is from a study comprising 14 patients with unifocal extranodal HS [26]. From this series surgical resection is the mainstay of treatment. Adjuvant radiation therapy may help reduce local recurrence rates. In cases of disseminated disease chemotherapy should be used but its role in the adjuvant setting for unifocal disease remains unclear. The optimal chemotherapy regimen remains unclear and patients are mostly treated with lymphoma based treatments such as cyclophosphamide, doxorubicin, vincristine, prednisone (CHOP) or CHOP and etoposide. In our six cases, five received chemotherapy with their initial treatment due to advanced disease. In our case with brain involvement, high dose methotrexate based treatment was prescribed similar to treatments used in primary central nervous system lymphoma. In the other cases, chemotherapy was similar to that used in large cell lymphoma including CHOP and CHOP like therapy with etoposide. The ideal treatment for multifocal disease in patients with HS remains unclear and patients should be referred for clinical trials or treatment at tertiary care centers.

### 3.3. Follicular Dendritic Cell Sarcoma

FDCS is a very rare clonal neoplasm of follicular dendritic cells (FDCs). Follicular dendritic cells are derived from stromal cells located in the follicles of activated lymph nodes. These cells accumulate and entrap immune complexes and store and retain antigens for a long time serving as a nidus for B-cell proliferation and differentiation along with help from T-cells [31,32,33]. Since abnormal FDCs can be seen in other disorders, it has been thought that there may be a link between disorders of FDCs and FDCS. Interestingly, one of our cases was a case of FDCS that was thought to arise in lymph nodes harboring dysplastic FDCs in Castleman’s disease which may have occurred through trans-differentiation leading to aclonal expansion of FDCs as suggested in the literature [34,35]. FDCS and non-neoplastic FDCs of Castleman’s disease both express epidermal growth factor receptor (EGFR) which may promote FDC persistence leading to FDCS [36]. FDCS has also been associated with follicular lymphoma likely through trans-differentiation of the follicular lymphoma [28].

The presentation of FDCS has been reported to have a mean age of 44 years in two series [4,37]. In cases of localized FDCS there is usually a benign course with median survival reported in a large series to be 168 months (range 2–360 months) with risk of local recurrence and distant metastasis of 27%–28% [38]. Larger tumor size (≥6 cm), presence of coagulative necrosis, high mitotic count (≥5 per 10 high power fields), and cytological atypia are associated with poor prognosis [38,39,40]. Presentation of disease in half of patients will be with a local cervical lymph node or intra-abdominal mass similar to one of our cases [38,41]. Although rare, extranodal involvement is mainly seen in the liver, lung, tonsils, or spleen [38]. In our patient with Castleman’s disease, multiple lymph nodes were enlarged and this was mainly thought to be due to his Castleman’s disease and not FDCS.

**Figure 3:**
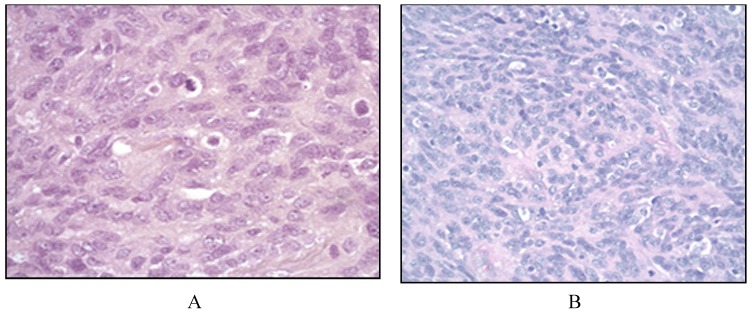
Follicular dendritic cell sarcoma. Histologically, there is effaced architecture by a spindle cell proliferation. The spindle cells form fascicles and whorls (**A**). On closer magnification, the neoplastic cells show admixed spindled to ovoid nuclei with indistinct cytoplasmic borders, mitosis and mild pleomorphism. Rare small lymphocytes are seen in the background.

In patients suspected with FDCS workup should be similar to that of patients with suspected lymphoma, including a comprehensive physical examination, CT scans from the neck to the pelvis with contrast. The benefit of bone marrow biopsy in these patients, especially with unifocal disease is unclear. Histopathology is characterized by spindled to ovoid cells forming fascicles, whorls, diffuse sheets or nodules. Nuclear pseudo-inclusions are common and bi-nucleated and multinucleated tumor cells are seen (Figure 3) [2]. In FDCS, CD21, CD23, CD35 are positive while there is variable expression of CD68 [31,39,42].

Surgery remains the most common treatment with a Surveillance, Epidemiology, and End Results (SEER) study reporting 94% of patients with localized FDCS receiving surgical resection as initial treatment [41]. No benefit has been seen for adjuvant radiation therapy in patients with localized FDCS [38,41]. In our case with concurrent Castleman’s disease, the tumor was resected and the patient received no adjuvant therapy. Similar to our case with extensive disease, patients treated with combined chemotherapy and radiotherapy (*n* = 23) had excellent survival with only two deaths due to disease [38]. These data suggest the importance of combination chemotherapy and radiation therapy in advanced FDCS. Optimal treatments in FDCS is unknown but regimens designed for aggressive lymphomas such as CHOP, ifosfamide, carboplatin, etoposide (ICE), and adriamycin, bleomycin, vinblastine, and dexamethasone (ABVD) have been used with varying success [31,38,43,44]. These patients should be treated on clinical studies when possible and should be referred to tertiary care centers.

### 3.4. Interdigitating Dendritic Cell Sarcoma

Normal interdigitating dendritic cells (IDCs) present antigens to T cells located in the paracortex and regulate cellular immune response [38,45,46,47,48,49]. IDCS are clonal disorders of IDCs and have been reported to be associated with other hematologic and solid tumor malignancies including B-cell neoplasms, mycosis fungoides, and neoplasms of the skin, liver, stomach, colon, breast, and brain [38]. A clonal relationship between IDCS and low grade B cell lymphomas including chronic lymphocytic leukemia with high risk features such as the 17p deletion has been postulated [1,28,50]. Neither of our cases had another associated malignancy.

Median age at diagnosis is reported to be 56.5 years (range 21 to 88 years) with a male predominance. Patients with higher stage, younger age, and intra-abdominal involvement have been shown in univariate analysis to have a worse prognosis [38,41]. Median survival for patients with disseminated IDCS has been reported to be 9–10 months while those with localized disease did not reach a median survival in two reported series [38,41].

Commonly solitary lymph node, skin, and soft tissue masses have been described in the literature similar to the presentation of both of our cases [2,42,46,51,52,53]. Staging should at least include CT scans to rule out other sites of disease and bone marrow aspirate and biopsy if clinically indicated.

**Figure 4:**
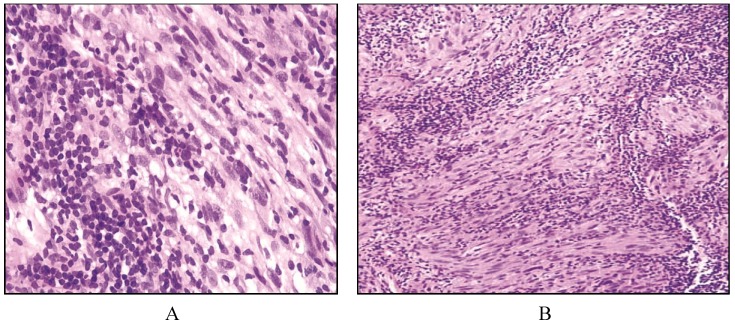
Interdigitating dendritic cell sarcoma. (**A**) Sinusoidal pattern by large neoplastic cells with elongated nuclei, copious pink cytoplasm, and indistinct cell borders with rare mitosis; (**B**) On high magnification, the spindle cells show mild to moderate nuclear atypia, hyperchromatism and mild pleomorphism with sheets of small lymphocytes.

Histologically, large spindle to ovoid cells with formation of whorls are seen with pink cytoplasm and indistinct cell borders. Typically, sheets of small lymphocytes intermingling with the large histiocytic cell population is a key diagnostic feature (Figure 4). This finding is less typical of carcinomas or sarcomas unless the carcinomas are of lymphoepithelial origin [42]. Immunophenotype includes CD21 negative, CD23 negative and CD1a negative, S100 positive, CD45 positive and have variable CD68 positive tumors [4,51].

Surgical resection remains the main treatment although the effect of surgery on overall survival is controversial with one study reporting a survival benefit while another showed that there was no difference between surgery and radiation treatment for localized IDCS [38,41]. Currently, either surgical resection or radiation therapy is recommended for localized IDCS. In disseminated disease, chemotherapy has been used and regimens such as CHOP, ICE and ABVD have promising results [38,52,54]. Patients should be treated on clinical trial or referred to tertiary care centers for treatment of this rare disorder. 

### 3.5. Indeterminate Dendritic Cell Sarcoma

INDCSs are extremely rare neoplasms of indeterminate cells and have only been reported in case reports. Indeterminate cells share morphologic and immunophenotypic features with Langerhans cells (except there are no Birbeck granules on electron microscopy in INDCS) which has led to speculation that indeterminate cells represent a mature form of Langerhans cells [55,56,57,58]. Associations between indeterminate cells and disorders such as nodular scabies, pityriasis rosea and low grade B-cell lymphomas have been reported [59,60,61,62,63]. Most cases report that patients present with one or more papules, nodules, or plaques on the trunk, face, neck or extremities similar to our case of INDCS [64,65]. Diagnosis is usually made by skin biopsy of a lesion. In cases of localized disease further work up with systemic CT scans and bone marrow biopsy is not indicated.

**Figure 5:**
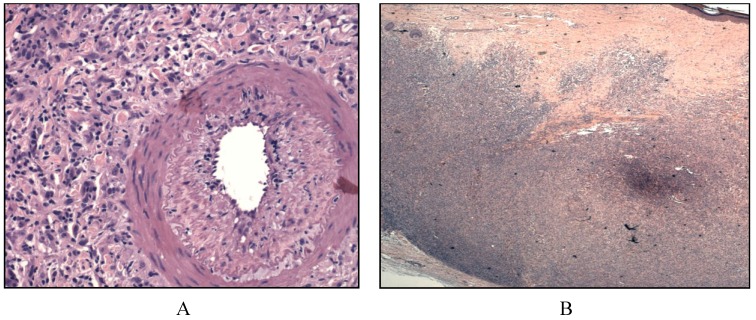
Indeterminate dendritic cell sarcoma: (**A**) Low power view showing deep diffuse dermal tumor nodule extending to subcutis; (**B**) High power shows the histiocytic infiltrate with spindly to dendritic to polygonal cells with oval to pleomorphic nuclei, abundant pink cytoplasm adjacent to a hypertrophic arteriole.

Histologically, dermal lesions are diffusely infiltrating and are composed of cells with irregular nuclear grooves and clefts that resemble Langerhans cells (Figure 5) [65]. These cells lack Birbeck granules on electron microscopy and desmosomes are lacking but interdigitating cell processes can be present [2,64]. Immunohistochemistry is S-100 positive, CD1a positive, and are negative for CD21, CD23, CD35, langerin, and B and T cell markers [64]. 

Surgical resection is the therapy of choice of INDCS and many lesions can spontaneously regress [59,64]. The role of chemotherapy and radiation therapy remains unclear in cases of disseminated INDCS.

### 3.6. Fibroblastic Reticular Cell Tumor

FRCTs are rare neoplasms of fibroblastic reticular cells which are stromal support cells located in the parafollicular areas and the deep cortex of lymph nodes. FRCTs mainly present in the lymph nodes but can occur in the spleen, lung, liver, and soft tissue [38,66]. 

In a pooled analysis of 19 patients with FRCT it was reported that the median age was 61 years with a male predominance [38]. The majority of patients in this study (16/19) presented with nodal disease with cervical and mediastinal lymph nodes being the most common sites of presentation. Patients with localized disease had a two year survival rate of 85.7% with median survival not being reached [38]. Patients with distant disease died in 2 years with a median survival of 13 months [38]. Systemic workup for disseminated disease with CT scans, bone marrow biopsy in patients with FRCT in one lymph node is unclear and should only be considered in cases that present with multiple enlarged lymph nodes.

Morphologically FRCT cells are spindle to ovoid cells with whorls in the paracortical areas associated with abundant reticulin staining fibers. Immunohistochemistry is positive for Factor XIIIa, desmin, vimentin, and smooth-muscle actin and can have variable cytokeratin expression [2,67]. 

Treatment for FRCT should be determined on a case by case basis. In those with localized disease surgical resection is likely adequate [38,66]. In our case, although the patient had involvement of multiple lymph nodes, he was observed without any worsening of disease at 51 months of follow up suggesting an indolent course. 

## 4. Conclusions

Although our study has the inherent limitations of retrospective case series, including limited immunohistochemical staining to confirm histological diagnoses, our experience over the last 25 years with dendritic cell and histiocytic tumors highlights that the majority of patients with these disorders have limited involvement and have a long overall survival. Currently, surgical resection remains the initial treatment in the majority of cases where localized disease is present. The role of adjuvant radiation or chemotherapy has not yet been determined in any of these disorders. In cases of disseminated disease multimodality treatment is likely needed to improve outcomes although data is limited. 

Being a tertiary care referral center, many of our cases received staging with CT scans, bone marrow biopsy and aspirate, and had multisite disease indicating more advanced disease. It is likely that we are underestimating the number of cases of these disorders in our region since many patients with skin only involvement or localized disease were likely treated by dermatologists or other specialists and were not captured by our search. These neoplasms remain difficult to diagnose even with advances in immunohistochemistry and since the majority of our cases were either diagnosed prior to the 2008 WHO classification or did not have additional tissue for further immunohistochemistry we were unable to determine the full immunohistochemical profile in all the cases. Due to the rarity of these disease we suggest a combination of surgical pathology with hematopathology review, full immunohistochemical profiles, and treatment at tertiary care centers to better manage these rare, confusing and “orphan” diseases. Although we were unable to make any conclusions with our series of patients, we feel that by reporting our data it may encourage others to report and collaborate with us in specializing in these rare disorders. With improved reporting on these rare diseases it may be possible to better study each of these disorders in order to improve outcomes in adult patients with LCH, HS, FDCS, IDCS, INDCS, and FRCT. 
